# Carbon Emissions Trading and Sustainable Development in China: Empirical Analysis Based on the Coupling Coordination Degree Model

**DOI:** 10.3390/ijerph18010089

**Published:** 2020-12-24

**Authors:** Jingru Huang, Jie Shen, Lu Miao

**Affiliations:** 1 School of Economics, Shenzhen University, Shenzhen 518060, China; Jrhuangs@163.com; 2 Shenzhen Audencia Business School—Shenzhen University, Shenzhen University, Shenzhen 518060, China; Jie.shen@szu.edu.cn; 3 China Center for Special Economic Zone Research, Shenzhen University, Shenzhen 518060, China

**Keywords:** emissions trading scheme (ETS), sustainable development, coupling coordination degree (CCD) model

## Abstract

Despite the extensive attention paid to emissions trading scheme (ETS) approaches, few studies have examined whether such ETS policies can lead to sustainable development in China. Drawing on the ideas of coupling and synergistic development, this study views sustainable development as the result of the interactions between the economy and the environment and constructs an index system to measure economic development and environmental quality. The system coupling model is used to reflect the synergistic interactions between the economy and the environment systems. The coordination degree model is then used to assess the economic–environmental coupling coordination degree in order to measure sustainable development. The empirical results show that the ETS can help in achieving economic–environmental sustainable development in the pilot cities. Moreover, the better the socioeconomic development of a city, the better effects of the ETS on sustainable development. However, it is more difficult to achieve economic–environmental coordinated development in industrially developed areas (e.g., Guangdong). These findings provide empirical evidence that the market-based ETS could alleviate the conflict between economic development and environmental pollution and could help in achieving sustainable development in emerging economies.

## 1. Introduction

Since the advent of the industrial revolution, the global economy has been booming; however, the negative impacts of this economic development have become more prominent. The contradiction between economic growth and environmental protection has become a major concern. After more than 40 years of reform and opening-up, China has experienced miraculous economic growth [1], becoming the world’s second largest economy. However, behind this rapid economic development, the long-standing extensive growth approach has put pressure on the environment, which is close to a critical threshold; China has also become the world’s largest emitter of SO_2_ and CO_2_ [2]. As the largest developing country, the Chinese government recognizes the significance and urgency of environmental protection and has launched a comprehensive sustainable development strategy to deal with it. Initially, China addressed environmental pollution with laws and policy measures, and then introduced a market-based emissions trading scheme (ETS) for emissions reduction. Currently, using market mechanisms for environmental pollution control has become an important strategy for eco-environmental protection, and the Chinese government is vigorously promoting the establishment of a nationwide carbon emissions trading (CET) system. However, whether the ETS can achieve sustainable development has not been confirmed in theory or empirical research.

As one of the mainstream mechanisms for the international community to deal with climate change [3], various ETS approaches have attracted a lot of attention from scholars and numerous research studies have accumulated. Some studies have compared and contrasted the design of mechanisms such as the approaches of allocation [4,5] and trading prices [6] in terms of emissions rights. Other studies have constructed evaluation models to assess the policy effects of ETS, such as the emission reduction effect [7] and technological innovation effect [3,8]. Despite an abundance of literature, carbon trading systems have economic and environmental dividends and can even lead to win–win situations. (e.g., Hou et al. [9]; Ren et al. [8]). However, the relationship between economic development and environmental quality is the result of the complex interactive impacts of the two systems. For instance, Krueger’s study confirmed an “inverted U-shaped” relationship between economic growth and pollution emissions [10,11], known as the environmental Kuznets curve (EKC), which has been widely accepted [12]. However, subsequent scholars have verified various relationships between the two, for example Friedl and Getzner [13] found an “N-shaped” relationship between economic growth and CO_2_ emissions in Austria over the period 1960–1999. This finding resurfaced in Onafowora and Owoye’s [14] study. The research by Zhu et al. [15] confirmed an inverted “N” relationship between industrial emissions and GDP per capita. With nitrogen dioxide (NO_2_), respirable particulates (PM), and carbon monoxide (CO) as the main indicators of air pollutants, the relationship between Beijing’s economic development and air quality shows a “U-shape” [16] Specifically, coordinated development does not mean “equal development”, but rather mutually reinforcing, coupling, and synergistic development [17], which can lead to sustainable development [18]. Sustainable development emphasizes the balanced development of the economy, society, and the natural environment [19,20], which refers to the process of synergistic integration, interaction, and co-development of economic, social, natural, and environmental subsystems [21]. Based on this, we argue that previous studies exploring the impact of ETS approaches on economic growth and pollutant emissions separately do not adequately reflect the impacts of ETS approaches on development sustainability. There is a lack of empirical research on whether the ETS can alleviate the contradiction between economic growth and the environment that China is currently facing, and whether it can lead to coordinated economic and environmental development in order to ultimately promote sustainable development.

Therefore, this study constructs an index system for measuring economic development and environmental quality and uses the system coupling model, defining the degree of interaction between economic and environmental elements as the economic–environmental coupling degree, which reflects the synergistic result of the interactions between economic and environmental systems, ranging from disorder to order. The coordination degree model is then used to assess the economic–environmental coupling degree, and the level of sustainable development is measured by the degree of coupling coordination. The contribution of this study, in contrast to previous work, is the use of the theory of system coupling and coordination in physics and the idea that the interactions of elements in a system determine the ultimate direction of the system [22,23]. The impact of the ETS on the level of coordinated economic and environmental development is discussed by including economic and environmental systems as subsystems of sustainable development, emphasizing the impact of the ETS on the sustainability of regional development.

## 2. Literature

Previous studies on ETS approaches have mainly focused on policy design and evaluation. In terms of policy design, the first step is to determine the emissions cap. Pang & Duan [24] conducted a comparative study of cap setting approaches in seven ETS pilots in China and concluded that flexible caps, based on the aggregated allocation of allowances to participants, can reduce emissions and uncertainty related to the resulting costs. After setting an emissions cap, policymakers need to choose how to allocate it. The results of Yu et al. [25] study, based on a computable general equilibrium (CGE) model, showed that the initial allocation of allowances would have a significant impact on the size of the carbon market and the trading behavior of the industry. The power generation and oil sectors would bear the largest output losses, while the metal smelting sector would be the main seller. Currently, the most common approaches for allocating allowances are auctions or free allocation of allowances. In the initial phase of the ETS, the free allocation approach was easy to implement [26]; however, it was gradually replaced by auctions [4]. In practice, the main methods of free allocation of allowances are grandfathering based on historical emissions (emissions intensity) and industrial benchmarks that differentiate allocations based on the nature of a product or the production process [4,5]. Moreover, by constructing an allocation equality assessment model, Ye et al. [27] found that the benchmarking mechanism can produce a more even sharing of the burden of carbon emissions reductions across provinces and can reduce uncertainty. Therefore, the benchmarking mechanism is a suitable alternative to the current equitable provincial allocation of carbon allowances in China. The benchmarking allocation method is being used more frequently, however the free allocation for a single company is considered based on a combination of historical emissions, industry characteristics, and product properties [5]. Regarding the trading price of carbon emissions, Jotzo and Löschel [28] believes that this mainly depends on the underlying emissions growth rates. Wang et al. [6] designed two cap-and-trade policy scenarios for the Guangdong ETS. From the simulation results, while both policies produced emission reduction effects, they found that a tighter carbon constraint would lead to a higher carbon price, and thus a greater loss of sectoral output. In addition, regulatory mechanisms are an important part of maintaining the operation of the emissions trading system. Ye et al. [4] pointed out that the regulatory system for China’s seven pilot carbon markets suffers from uncertainty regarding the responsibilities of regulatory agencies, as well as inadequate regulatory systems and policies. In this regard, they proposed strategies to strengthen the legal effectiveness of carbon market regulation, establish a dedicated regulatory body, and encourage the participation of public and industry associations to form an external oversight mechanism.

Previous studies have examined the impacts of ETS policy implementation on emissions reductions, economic performance, technological innovation, and productivity. In terms of emissions reductions, Hou et al. [9] showed that the 2007 SO_2_ ETS pilot policy in China significantly reduced SO_2_ emissions and SO_2_ intensity, consistent with Wu et al. [29] findings. Zhang et al. [30] pointed out that although the carbon ETS in China is still accompanied by an immature market environment, inadequate infrastructure, limited emissions trading, and low market liquidity, it has had a significant effect on China’s carbon emissions reductions in recent years. Zhang et al. [31] concluded that China’s ETS significantly reduced the carbon emissions of the industrial subsector in the pilot area, and this impact showed an increasing trend. Moreover, Yan et al. [7] results showed that based on city-level data, the ETS in China did have a significant “reduction effect” on haze pollution levels, verifying the collaborative governance effects of the ETS on air pollution. This possibly occurred through “boosting the application and transformation of green technologies among enterprises” and “transferring heavily polluting industries”. Some scholars have studied environmental performance and economic performance together. For example, Wang et al. [32] and Ren et al. [8] showed that China’s carbon trading system can achieve both environmental and economic “win–win” outcomes, whereas Dong et al. [33] applied the difference-in-difference (DID) method and the improved DEA model to test the impacts of the ETS on the economy and environment, finding that the ETS significantly reduced carbon emissions but failed to increase GDP in the short term. However, in the long term, a carbon trading system can provide both environmental and economic dividends. This finding is consistent with the finding of Hou et al. [9]. Regarding the impact of ETS on technological innovation and productivity, the study by Ren et al. [8] based on enterprise data from 2004–2015 in China found that the numbers of patent applications and environmental patent applications in regulated enterprises in SO_2_ ETS pilot areas increased by 28.3% and 22.3%, respectively, compared to enterprises in non-pilot areas. At the same time, the policy performance was better in areas where environmental enforcement was more stringent. Jaraite and Maria [34] studied the power generation sectors in 24 EU countries from 1996–2007 and found that ETS approaches promoted technological innovation. Zang et al. [3] discussed the impacts of the EU ETS on industrial structural upgrading in the member states of the European Union (EU). They found that the implementation of the EU ETS greatly contributed to the upgrading of the country’s industrial structure, with the policy effect gradually increasing over time. Applying the DID model and parallel multiple mediator model, Chen et al. [35] explored the abatement effects of the carbon ETS and the influencing paths of the policy at both national and regional levels.

ETS, as the main market-based environmental regulatory tool in China, is also important for achieving sustainable development. As we mentioned before, however, unilateral reduction of pollutant emissions or economic growth cannot be considered sustainable development. Whether ETS can contribute to the harmonious development of the economy and the environment needs to be tested through empirical studies. Therefore, from the perspective of coupling and synergistic development, this study views sustainable development as the result of the interaction between economy and environment and discusses the impacts of ETS approaches on regional sustainable development.

The remainder of the paper is organized as follows. Section 3 introduces the case study for the paper. Section 4 outlines the methodology and data. The empirical results are reported in Section 5. Finally, Section 6 concludes the paper with a discussion.

## 3. Case Study

China was the first developing country to launch a climate change program and has made comprehensive sustainable development a national strategy. In the early days, China adopted laws and regulations to deal with climate change, such as the “Air Pollution Prevention and Control Law of the People’s Republic of China”. In 2002, China began exploring and practicing emissions trading mechanisms. In October 2011, the Chinese government designated Beijing, Tianjin, Chongqing, Shanghai, Shenzhen, and the provinces of Hubei and Guangdong as the pilot regions for carbon emissions trading schemes, then in 2017 released the National Emissions Trading Market Construction Plan (for the electricity sector), marking the establishment of the first large-scale national market for the trading of emissions rights in China. So far, emissions trading has become an important instrument in reducing pollution and protecting the environment in China.

In the research based on the data from the carbon emissions trading (CET) pilot cities in China since 2011, the coupling coordination degree (CCD) model has been used to quantify the level of economic–environmental coordination in the pilot regions from 2008–2018 and to explore the impact of CET on sustainable development. Of the seven pilot regions, four cities are directly under control of the central government. These are Beijing, the capital of China, and the political and cultural center; Tianjin, near Beijing, which is the largest industrial center in northern China; Shanghai, a metropolis and base for finance, trade, shipping, and technological innovation; and Chongqing, the economic center and manufacturing base of southwest China. The scheme also includes the provinces of Guangdong and Hubei. Guangdong is China’s largest economic province, with a GDP exceeding 10 trillion (2020 China Statistical Yearbook) and is the window to the outside world in terms of economic, cultural, scientific, and technological fields. Hubei is an important transportation hub in central China and a base for the optoelectronic industry and equipment manufacturing. There is also China’s special economic zone, the city of Shenzhen, which is the largest financial and technology industry innovation center in southern China.

## 4. Methodology

### 4.1. The Indicator System for Evaluation of Economic Development and Environmental Quality

Based on the previous literature (e.g., Li et al. [23]; Wan et al. [36]; Li et al. [20]; the World Bank World Development Indicators [37]) and the availability of data, we developed a system of indicators for economic development and environmental quality assessment. The specific indicators are shown in Table 1.

### 4.2. Data Pre-Processing

The data used were from the China Urban Statistical Yearbook (2009–2019), while the missing values were replaced with the average values for the cities from 2008–2018. Due to the different dimensions, magnitudes, and positive and negative orientations of the indicators, this study standardized the data using Equations (1) and (2).

(1) $$y_{ij}=\frac{x_{ij}-min\left(x_{ij}\right)}{max\left(x_{ij}\right)-min\left(x_{ij}\right)}\mathrm{Positive}\;\mathrm{indicator}\;$$

(2) $$y_{ij}=\frac{max\left(x_{ij}\right)-x_{ij}}{max\left(x_{ij}\right)-min\left(x_{ij}\right)}\mathrm{Negative}\;\mathrm{indicator}$$

where $y_{ij}y_{\mathrm{ij}}$ is the normalized value; $x_{ij}$ represents the initial value of indicator *j* for city i; $max\left(x_{ij}\right)\;\mathrm{and}\;min\left(x_{ij}\right)$ represent the maximum and minimum values for indicator j for city i. Equation (1) was chosen for calculations when higher indicator values were better (positive indicator); Equation (2) was used instead (negative indicator).

### 4.3. The Entropy Weight Method (EWM)

The entropy weight method was developed from the Shannon entropy, which is a quantitative measure of the uncertainty of information systems [38]. Compared to subjective human evaluation, the entropy weight method is an objective weighting method that has higher reliability, as its weighting coefficient depends entirely on the value of the indicator. [36,39]. The specific calculation steps are as follows [23]:

The proportion of the indicator *j*: $p_{ij}=\frac{y_{ij}}{\sum_{i=1}^{m}y_{ij}};$

Information entropy of the indicator:$\;e_{j}=-\frac{1}{\ln m}\sum_{i=1}^{m}p_{ij}\times \ln y_{ij}\left(0\leq e_{j}\leq 1\right)$;

Entropy redundancy: $d_{j}=1-e_{j}$;

Weight of the indicator: $w_{j}=\frac{d_{ij}}{\sum_{i=1}^{n}d_{ij}}$;

Evaluation of a single indicator: $s_{ij}=w_{j}\times y_{ij}$;

Evaluation of subsystems: $s_{j}=\sum_{j=1}^{n}s_{ij}$.

### 4.4. The Coupling Coordination Degree Model (CCDM)

Coupling, a concept encountered in physical electronics [40], is a phenomenon in which two or more systems interact and influence each other [22,23]. The coupling coordination degree (CCD) indicates the magnitude of the degree of benign coupling of interactions between systems or elements, reflecting the excellent state of coordination between systems or elements [41]. The coupling coordination degree model (CCDM) is a widely used approach in the study of the interaction and coordinated development between different subsystems [20], such as between urbanization and an ecosystem (e.g., Liu et al. [22]; Li et al. [23]). In this study, the coupling coordination degree between economic development and environmental quality was constructed as follows [22,42]:

$$C=2\times {\left\{\left(U_{1}U_{2}\right)/\left[\left(U_{1}+U_{2}\right)\left(U_{1}+U_{2}\right)\right]\right\}}^{\frac{1}{2}}$$

where C is the degree of coupling, with a range of [0, 1]. U_1_ and U_2_ represent the economic development and environmental quality, respectively.

$$\begin{array}{c} D={\left(C\times T\right)}^{\frac{1}{2}}\\ T=\left(\alpha U_{1}+\beta U_{2}\right) \end{array}$$

where D represents the degree of coordination, with a range of [0, 1]. The closer D is to 1, the better the coupling coordination level. T is a comprehensive coordination index of economic development and environmental quality, reflecting the impact of their overall level on coordination [23,36], with a range of [0, 1]. The coefficients of α and β, which refer to the contributions of the economy and the environment, draw on Yu et al. [41] study, with α and β values of 0.5, respectively.

## 5. Results

**Figure 1:**
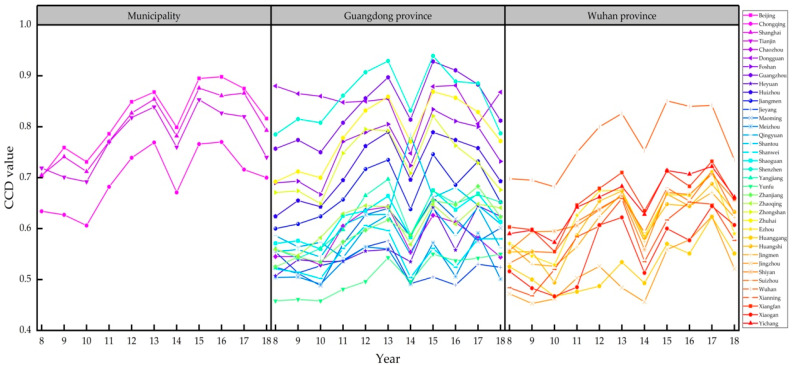
Changes in coupling coordination degree (CCD) value for the cities over the period 2008–2018.

First of all, based on the EWM and the CCDM, we calculated the economic–environmental coupling coordination degree of 37 pilot cities that implemented carbon emissions trading policies from 2011–2018. The values and trends are shown in Table A1 and Figure 1, respectively. According to the descriptive statistical results based on the CCD values of the pilot cities (as shown in Table 2), we found that in 2008–2010, prior to the issuance of the carbon emissions trading policy, the CCD values ranged between 0.4–0.9, implying that the levels of economic–environmental coordinated development in the pilot cities ranged between “endangered disorder” and “good coordination”. In 2011–2018, after the policy was issued, the CCD values of the 37 pilot cities ranged between 0.4–1.0, meaning that the levels of economic–environmental coordinated development ranged between “endangered disorder” and “extreme coordination”. At the same time, the mean CCD value for the pilot cities after introducing of the policy increased from 0.59–0.67, indicating that the carbon trading policy improved the coordinated development of the pilot cities.

Secondly, by calculating the growth rates for coupling coordination from 2011–2018 [12], we found that the growth rates in Tianjin, Chaozhou, Foshan, Huizhou, Jiangmen, Jieyang, Meizhou, Shaoguan, Shenzhen, Zhongshan, Zhuhai, Ezhou, Wuhan, and Xianning were negative (shown in Table 3). Of the 14 cities with negative growth, 10 were from Guangdong Province. The main reason for this was the rapid economic and industrial development in Guangdong, which led to greater pressure on the natural environment. Since the reform and opening-up in 1987, Guangdong has become one of China’s major economic provinces, especially in terms of industrial development, and is considered the “factory of the world” [43]. In 2019, Guangdong became the first province in China to exceed 10 trillion GDP, while its industrial value also ranked first in the country. This rapid industrial development has led to more serious environmental problems, and against this background it is more difficult for Guangdong to achieve harmonious economic and environmental development compared to other provinces and regions.

**Figure 2:**
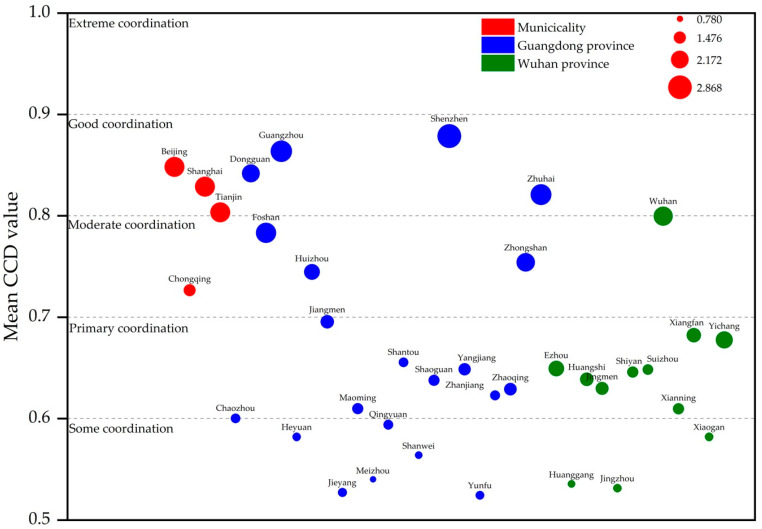
CCD levels of the pilot cities.

Thirdly, in this study, the average CCD value for each city from 2011–2018 was used to represent the overall level of sustainable development, with the values shown in Table 3. Drawing on the study by Shu et al. [44], the CCD of the pilot cities was classified into 10 grades (shown in Table A2), as shown in Figure 2. Eight cities, including Beijing, Shanghai, Dongguan, Tianjin, Shenzhen, Guangzhou, Zhuhai, and Wuhan, had a good coordination level for CCD, accounting for 21.6% of the total pilot city. Most of these cities are municipalities directly under control of the central government and provincial capitals. Cities with average CCD values at a moderate coordination level included Foshan, Zhongshan, Huizhou, and Chongqing (accounting for 10.8%), which showed better economic development. A total of 16 were classified as primary coordination cities, accounting for 43.2%, while 9 cities were classified as having “some coordination”, accounting for 24.3%. In addition, the bubble size in Figure 2 is determined by the city’s per capita GDP (logarithm), which can reflect the level of social and economic development of a region. Based on this, we concluded that the majority of cities’ economic–environmental coordination development was at the level of “primary coordination”; the better the level of economic and social development, the higher the economic–environmental coordination degree of the city.

## 6. Conclusions and Discussion

**Figure 3:**
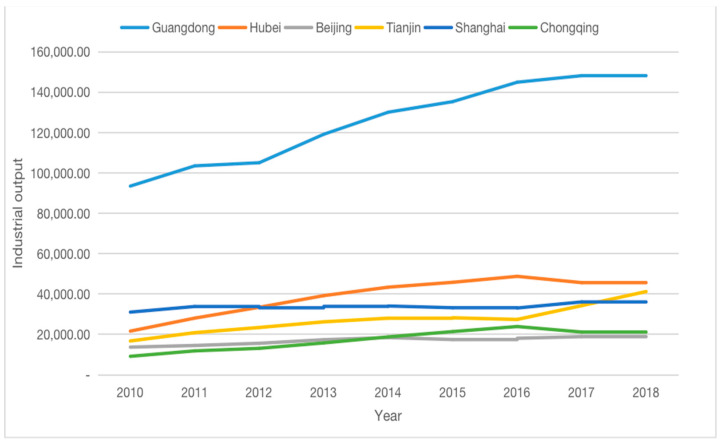
Industrial output value of the pilot regions and provinces from 2010–2018.

Through the calculation and analysis of the CCD values for the carbon trading pilot cities, our study found that the average CCD value for the pilot cities increased from “some coordination” to “primary coordination” after the implementation of the policy, indicating an increase in the level of sustainable development. In industrially developed areas (defined by the total of industrial output value), environmental pollution was relatively serious; for example, in Guangdong Province which industrial output value is much higher than that of other pilot region and province (showed in Figure 3), there were more cities with a negative CCD growth rate, indicating that it would be more difficult to coordinate economic and environmental development. Most cities were at the level of primary coordination, while the more economically developed areas had better CCD values and better levels of sustainable development. Finally, the ETS pilot policy had a positive effect on the coordinated economic–environmental development of cities.

Compared with existing studies, Wang et al. [32] study focused only on the impacts of ETS approaches on the development of a low-carbon economy, expressing the contributions of ETS approaches to the economy and environment in terms of GDP output per unit of carbon emissions. The coordinated development assessment index system constructed in this study covers more elements of the economy–environment system, reflecting more comprehensively the impacts of the ETS on the economy and environment, and the degree of coordinated development between the two. In studies on the impact of environmental regulation on coordinated development by Yuan et al. [45] and Zhang et al. [30], the indicator related to the cost of environmental governance was chosen as a proxy variable for environmental regulation, which differed from the actual intensity of environmental regulation and had a negative impact on the reliability of the results. This paper quantified the elements involved in the economic and environmental systems. The coupling coordination degree model can successfully reflect the degree of interaction and influence between the two systems, more accurately reflecting the level of coordination between economic development and environmental protection.

The policy implications of this study are as follows. First of all, the carbon ETS policy can promote harmonious economic–environmental development at the city level, and the Chinese government should continue to promote the ETS in order to achieve sustainable development. Secondly, in areas with more developed industries, the growth trend of sustainable development levels is not ideal. These regions should adjust their economic and industrial structures which is considered the main reason for the resource curse [46], increase their proportions of high-tech industries and service industries, and reduce their proportions of resource-dependent industries and high-polluting industries. Finally, the better the economic and social development of a region, the better the level of sustainable development. Policy-makers should carry out differentiated policy design based on the development situation in the regions where the policies are implemented, while the effectiveness of the policy implementation should be ensured and improved through the innovation of the existing ETS approach.

This study has some limitations that need further discussion in the future research. On the one hand, the study did not consider the issue of “carbon leakage”. The relationship between the stringency of environmental policies and pollution havens was explored and the pollution haven hypothesis was supported in the study by Sadik-Zada and Ferrari (2020) [47]. Whether the increase in the level of sustainable development in the pilot areas of carbon trading policies is influenced by pollution transfer is a very valuable research topic, which is worthy of further discussion. On the other hand, as previously mentioned, sustainable development is the result of the interaction of social, economic, and environmental systems, and conflicts can exist between the systems in the process of moving from disorder to order. For example, the interaction between authoritarian regime types and natural resource wealth has a strong negative impact on economic modernization [48]. The direction and mechanisms of the interaction between the systems can be used as a direction for future research.

## Figures and Tables

**Table 1:** Indicator system for economic development and environmental quality subsystems.

Subsystem	Index
Economic development	Per capita GDP (10,000 yuan)
	Amount of Foreign Capital Act (10,000 USD)
	GDP growth rate (%)
	The proportion of secondary industry output value (%)
	The proportion of tertiary industry output value (%)
	Per capita retail sales of consumer goods (10,000 yuan)
	Per capita passenger volume (10,000 capita)
	Per capita freight volume (10,000 ton)
Environmental quality	Volume of industrial waste-water discharged per 10,000 Yuan of GDP (Ton)
	Volume of industrial sulfur dioxide emissions per 10,000 Yuan of GDP (Ton)
	Volume of industrial smoke (dust) emissions per 10,000 Yuan of GDP (Ton)
	Ratio of industrial solid waste comprehensively utilized (%)
	Ratio of urban sewage treatment (%)
	Ratio of domestic harmless garbage treatment (%)
	Per capita green area (m^2^/capita)
	Ratio of green coverage of built-up areas (%)

**Table 2:** Descriptive CCD statistics 2008–2018.

Variable	Obs	Mean	Std. Dev.	Min	Max
CCD (2008–2010)	111	0.5942973	0.0988561	0.453	0.88
CCD (2011–2018)	296	0.6744223	0.1117855	0.456	0.939

**Table 3:** The growth rate and mean of CCD values for economic development and environmental quality.

City	Province	Growth Rate	Mean *	City	Province	Growth Rate	Mean *
Beijing	Beijing	0.55%	0.848	Yangjiang	Guangdong	1.29%	0.649
Chongqing	Chongqing	0.38%	0.727	Yunfu	Guangdong	2.05%	0.524
Shanghai	Shanghai	0.41%	0.829	Zhanjiang	Guangdong	1.22%	0.623
Tianjin	Tianjin	−0.56%	0.803	Zhaoqing	Guangdong	0.27%	0.629
Chaozhou	Guangdong	−1.44%	0.600	Zhongshan	Guangdong	−1.38%	0.754
Dongguan	Guangdong	0.34%	0.842	Zhuhai	Guangdong	−0.11%	0.821
Foshan	Guangdong	−0.72%	0.783	Ezhou	Hubei	−0.82%	0.649
Guangzhou	Guangdong	0.07%	0.864	Huanggang	Hubei	2.25%	0.536
Heyuan	Guangdong	2.35%	0.582	Huangshi	Hubei	1.12%	0.639
Huizhou	Guangdong	−0.04%	0.745	Jingmen	Hubei	1.47%	0.630
Jiangmen	Guangdong	−0.11%	0.695	Jingzhou	Hubei	0.51%	0.531
Jieyang	Guangdong	−0.37%	0.527	Shiyan	Hubei	1.18%	0.646
Maoming	Guangdong	0.78%	0.610	Suizhou	Hubei	0.37%	0.648
Meizhou	Guangdong	−0.93%	0.540	Wuhan	Hubei	−0.29%	0.800
Qingyuan	Guangdong	0.50%	0.594	Xianning	Hubei	−0.17%	0.610
Shantou	Guangdong	0.05%	0.655	Xiangfan	Hubei	0.29%	0.682
Shanwei	Guangdong	0.48%	0.564	Xiaogan	Hubei	3.59%	0.582
Shaoguan	Guangdong	−0.27%	0.638	Yichang	Hubei	0.45%	0.678
Shenzhen	Guangdong	−1.23%	0.879				

* Value is the average of CCD from 2011–2018.

## Data Availability

Data available on request due to restrictions privacy. The data presented in this study are available on request from the corresponding author. The data are not publicly available due to privacy.

## Appendix A

**Table A1:** The CCD values for economic development and environmental quality subsystems from 2008 to 2018.

City	2008	2009	2010	2011	2012	2013	2014	2015	2016	2017	2018
Beijing	0.702	0.759	0.731	0.786	0.849	0.868	0.799	0.895	0.898	0.875	0.816
Chongqing	0.634	0.627	0.606	0.682	0.739	0.769	0.671	0.766	0.77	0.716	0.7
Shanghai	0.705	0.741	0.712	0.771	0.827	0.854	0.782	0.876	0.861	0.866	0.793
Tianjin	0.719	0.701	0.692	0.77	0.818	0.839	0.76	0.853	0.827	0.82	0.74
Chaozhou	0.545	0.545	0.529	0.605	0.636	0.643	0.554	0.626	0.612	0.581	0.544
Dongguan	0.88	0.865	0.86	0.848	0.85	0.855	0.748	0.879	0.881	0.806	0.868
Foshan	0.69	0.693	0.667	0.771	0.789	0.805	0.724	0.834	0.811	0.8	0.732
Guangzhou	0.757	0.774	0.75	0.808	0.856	0.897	0.814	0.928	0.911	0.883	0.812
Heyuan	0.507	0.539	0.535	0.536	0.556	0.559	0.535	0.642	0.558	0.645	0.624
Huizhou	0.624	0.655	0.643	0.695	0.762	0.79	0.696	0.789	0.774	0.758	0.693
Jiangmen	0.6	0.609	0.624	0.657	0.717	0.735	0.638	0.746	0.686	0.732	0.652
Jieyang	0.554	0.513	0.527	0.538	0.564	0.576	0.492	0.505	0.489	0.53	0.524
Maoming	0.524	0.512	0.488	0.57	0.627	0.64	0.585	0.661	0.619	0.575	0.601
Meizhou	0.504	0.505	0.491	0.536	0.564	0.56	0.491	0.572	0.506	0.591	0.501
Qingyuan	0.586	0.564	0.573	0.543	0.601	0.627	0.549	0.641	0.586	0.642	0.562
Shantou	0.555	0.559	0.544	0.607	0.627	0.628	0.784	0.661	0.681	0.646	0.609
Shanwei	0.521	0.514	0.502	0.561	0.606	0.596	0.504	0.561	0.524	0.58	0.58
Shaoguan	0.571	0.576	0.56	0.625	0.635	0.664	0.583	0.675	0.637	0.669	0.613
Shenzhen	0.785	0.815	0.808	0.861	0.907	0.929	0.832	0.939	0.889	0.885	0.787
Yangjiang	0.56	0.542	0.561	0.598	0.665	0.697	0.587	0.674	0.649	0.667	0.652
Yunfu	0.458	0.461	0.458	0.481	0.496	0.543	0.496	0.55	0.537	0.542	0.55
Zhanjiang	0.525	0.544	0.534	0.574	0.597	0.617	0.587	0.655	0.647	0.683	0.623
Zhaoqing	0.559	0.547	0.582	0.629	0.645	0.643	0.569	0.647	0.609	0.648	0.641
Zhongshan	0.671	0.674	0.649	0.748	0.795	0.792	0.709	0.821	0.763	0.729	0.676
Zhuhai	0.692	0.712	0.7	0.778	0.832	0.859	0.771	0.869	0.857	0.829	0.772
Ezhou	0.571	0.546	0.529	0.626	0.674	0.675	0.585	0.667	0.665	0.712	0.59
Huanggang	0.525	0.5	0.467	0.476	0.487	0.534	0.493	0.57	0.551	0.623	0.551
Huangshi	0.554	0.555	0.494	0.587	0.654	0.674	0.582	0.648	0.644	0.688	0.633
Jingmen	0.568	0.53	0.526	0.564	0.624	0.669	0.575	0.668	0.644	0.671	0.622
Jingzhou	0.472	0.453	0.462	0.503	0.526	0.484	0.456	0.559	0.578	0.624	0.521
Shiyan	0.557	0.594	0.595	0.606	0.637	0.664	0.559	0.671	0.667	0.707	0.656
Suizhou	0.532	0.555	0.553	0.614	0.639	0.664	0.592	0.68	0.654	0.713	0.63
Wuhan	0.698	0.695	0.682	0.75	0.8	0.826	0.753	0.851	0.84	0.842	0.735
Xianning	0.484	0.468	0.52	0.584	0.605	0.659	0.535	0.617	0.652	0.648	0.577
Xiangfan	0.603	0.598	0.555	0.646	0.679	0.71	0.635	0.713	0.683	0.732	0.659
Xiaogan	0.516	0.483	0.467	0.485	0.607	0.622	0.513	0.6	0.577	0.644	0.607
Yichang	0.59	0.597	0.573	0.642	0.662	0.683	0.628	0.714	0.707	0.722	0.662

**Table A2:** CCD rankings.

Accidentally and Coordination Degree	Accidentally and Coordination Degree Grade	Accidentally and Coordination Degree Level
(0.0~0.1)	1	Extreme disorder
[0.1~0.2)	2	Severe disorder
[0.2~0.3)	3	Moderate disorder
[0.3~0.4)	4	Mild disorder
[0.4~0.5)	5	Endangered disorder
[0.5~0.6)	6	Some coordination
[0.6~0.7)	7	Primary coordination
[0.7~0.8)	8	Moderate coordination
[0.8~0.9)	9	Good coordination
[0.9~1.0)	10	Extreme coordination
